# Amoxicillin-induced pemphigus foliaceus

**DOI:** 10.1016/j.jdcr.2025.10.047

**Published:** 2025-11-04

**Authors:** Evan Mak, Steven Rivera, Craig Fisher, Nicholas Choe, Patrick Voorhees, Paul Hahn

**Affiliations:** a Uniformed Services University School of Medicine, Bethesda, Maryland; b Department of Dermatology, San Antonio Uniformed Services Health Consortium (SAUSHEC), San Antonio, Texas; c Department of Pathology, San Antonio Uniformed Services Health Consortium (SAUSHEC), San Antonio, Texas; d Department of Pathology, Walter Reed National Military Medical Center, Bethesda, Maryland

**Keywords:** autoimmune, blistering, bullous, drug rash, immunobullous, pemphigus foliaceus, pemphigus, PF

## Introduction

Pemphigus foliaceus (PF) is an uncommon autoimmune blistering disease characterized by superficial epidermal acantholysis and autoantibodies targeting desmoglein 1 (DSG1). Although typically idiopathic, PF can be drug-induced and often presents with widespread erosions that may mimic infectious or inflammatory dermatoses. Common drug culprits include thiols/sulfhydryls such as captopril and penicillamine, which comprise 40% of drug-induced pemphigus cases.^1^ Beta-lactams are a known cause of drug-induced pemphigus vulgaris but are not a well-reported cause of drug-induced PF. Typical clinical presentation includes variable degrees of erythema with superficial flaccid bullae and superficial erosions with overlying bran-like scale commonly in a seborrheic distribution. Histologically, PF demonstrates subcorneal acantholysis that can be difficult to distinguish from staphylococcal-scalded skin syndrome and bullous impetigo on routine hematoxylin and eosin-stained sections. Additional evaluation with direct immunofluorescence aids in the diagnosis. Treatment of drug-induced pemphigus centers around drug identification, discontinuation, and initiation of topical or systemic anti-inflammatory therapies depending on the degree of involvement. A prior review found that 87.8% of patients with drug-induced pemphigus resolved after drug discontinuation^1^ and 75% of patient required treatments such as topical or systemic corticosteroids in addition to drug discontinuation to resolve the lesions.

## Case report

**Fig 1:**
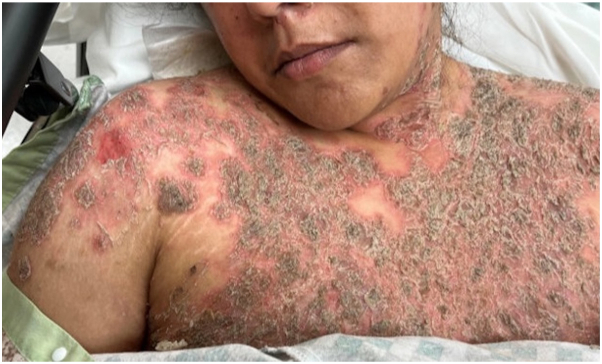
On admission, patient’s physical examination showed confluent erythematous tender plaques with overlying flaky scale. These lesions on her chest are representative of those elsewhere on her body.

A 30-year-old Fitzpatrick skin type III woman with no prior dermatologic history developed a progressive, painful blistering eruption 3 weeks after initiating amoxicillin for streptococcal pharyngitis. The eruption started on the trunk with subsequent spread to the scalp, face, neck, and proximal arms. No other new medications within the 6 months before onset were reported. Initially diagnosed as cellulitis, her condition worsened despite antibiotic therapy. Upon transfer to our hospital, examination revealed confluent erythematous tender plaques with overlying flaky scale, positive Nikolsky sign, and some intact fragile bullae affecting approximately 40% of her body surface area including the scalp, face, trunk, and proximal arms. No mucosal involvement was present (Fig 1).

**Fig 2:**
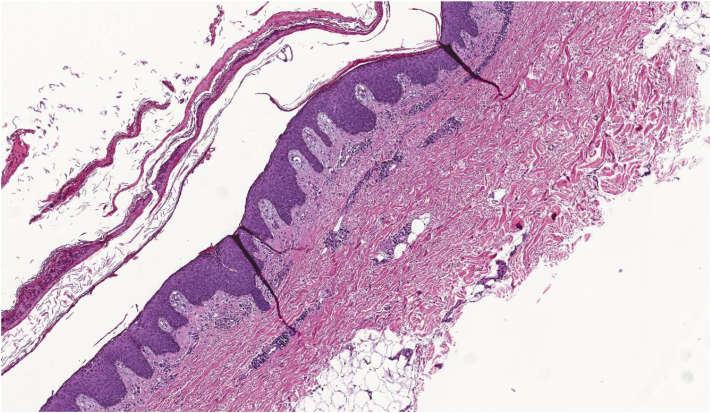
Physical examination 5 days after initiating treatment showed appreciable improvement of previously affected skin with healed erosions and re-epithelialized skin with minimal erythema.

**Fig 3:**
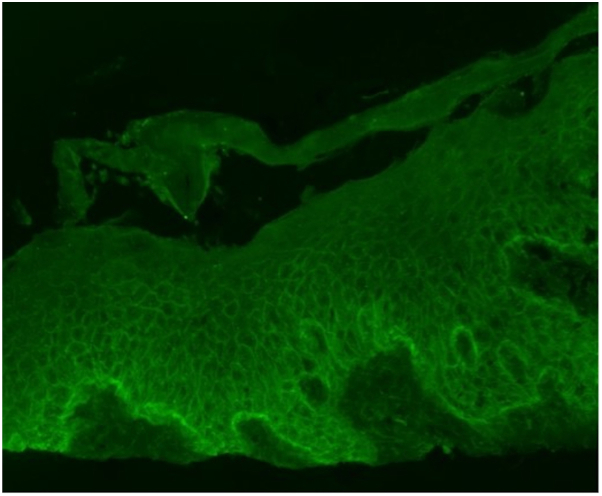
Histopathology supporting the diagnosis of pemphigus foliaceus. Hematoxylin and eosin showing foci of intragranular acantholysis with scattered hypergranulosis, intracorneal neutrophilic abscesses, and absent epidermal necrosis. In the dermis, there is perivascular lymphocytic inflammation with abundant neutrophils and eosinophils. Additionally, periodic acid–Schiff with fungus special stain is negative for fungal organisms. Gram special stain highlights cocciform bacteria within the serum crust.

**Fig 4:**
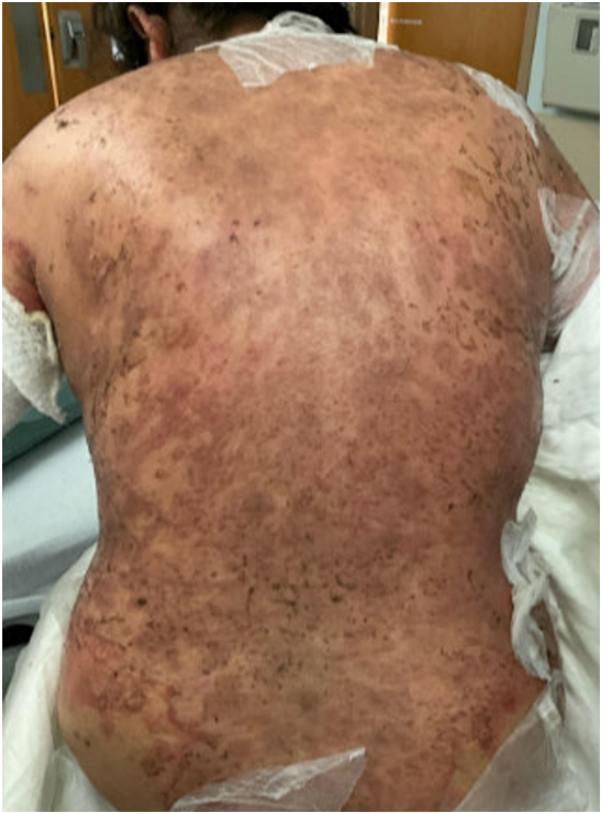
Direct immunofluorescence showed intercellular fluorescence is seen with antibodies to C3 and IgG (weak). C3 is also noted to have a linear staining pattern along the basement membrane.

A shave biopsy was performed that showed subcorneal acantholysis with neutrophils in the blister cavity (Fig 2). Serologic testing revealed markedly elevated anti-DSG1 antibodies. Her anti-DSG3 antibodies were slightly above the upper limit of normal. Bullous pemphigoid antigens 1 and 2 were negative. Direct immunofluorescence demonstrated intercellular fluorescence with antibodies to C3 and IgG, along with a linear C3 staining pattern long the basement membrane (Fig 3). Based on clinical, histologic, and serologic findings, a diagnosis of PF was made. Given the lack of prior dermatologic history and timing 3 weeks after starting amoxicillin her PF eruption was deemed to be most likely drug-induced. The patient responded well to oral prednisone monotherapy (Fig 4). Intermittently mild flaring of her cutaneous disease was noted in the 2 months after discontinuation of amoxicillin but her overall disease improved substantially with a trend toward clearance.

## Discussion

Drug-induced PF is an uncommon entity commonly presenting with diffuse superficially blistering and erosions with overlying flaky, bran-like scale in a seborrheic distribution. Common drug culprits include thiols/sulfhydryls, such as captopril and penicillamine,^1^^,^^2^ with beta-lactams such as amoxicillin rarely reported. Our case highlights a classic example of drug-induced PF secondary to a rare drug culprit, amoxicillin. The overall pathogenesis of drug-induced PF is similar to classic PF including the presence of IgG autoantibodies targeting DSG1,^3^ a cadherin component of desmosomes in the upper layer of the epidermis.^4^^,^^5^ In this case, the direct immunofluorescence showed a weak staining of the basement membrane with antibodies to IgG and C3. This same pattern of sections of weak basement membrane staining has been shown in PF biopsies documented by Karlhofer et al.^6^ Thiol drugs can bind keratinocyte proteins, altering their antigenicity and triggering an autoimmune response resulting in acantholysis,^2^ but the exact mechanism by which beta-lactams induce pemphigus has not been elucidated.^1^^,^^2^

Before the advent of corticosteroids, PF carried a high mortality rate. Today, it is treatable and often responsive to oral corticosteroids.^4^^,^^7^ First-line therapy for drug-induced pemphigus is drug discontinuation commonly with adjunct prednisone, with or without immunosuppressive adjuvants such as mycophenolate mofetil, methotrexate, dapsone, rituximab, or intravenous immunoglobulin.^4^^,^^7^ In our patient, drug discontinuation and prednisone monotherapy was effective, and adjunctive treatment was deemed unnecessary.

Our case highlights classic clinical and histopathologic features of PF while identifying amoxicillin as another possible cause of drug-induced pemphigus. It reinforces considering PF in patients with a widespread vesiculobullous eruption after penicillin use. Prompt recognition, drug identification, and early intervention with drug discontinuation critical in optimizing outcomes for patients with drug-induced vesiculobullous eruption.

## Conflicts of interest

None disclosed.
